# Data Resource Profile: Genomic data in multiple British birth cohorts (1946–2001)—linkage with health, social, and environmental data from birth to old age

**DOI:** 10.1093/ije/dyaf141

**Published:** 2025-08-18

**Authors:** Gemma Shireby, Tim T Morris, Andrew Wong, Nish Chaturvedi, George B Ploubidis, Emla Fitzsimmons, Alissa Goodman, Adelaida Sanchez-Galvez, Neil M Davies, Liam Wright, David Bann

**Affiliations:** Centre for Longitudinal Studies, University College London, London, United Kingdom; Centre for Longitudinal Studies, University College London, London, United Kingdom; Unit for Lifelong Health and Ageing, University College London, London, United Kingdom; Unit for Lifelong Health and Ageing, University College London, London, United Kingdom; Centre for Longitudinal Studies, University College London, London, United Kingdom; Centre for Longitudinal Studies, University College London, London, United Kingdom; Centre for Longitudinal Studies, University College London, London, United Kingdom; Centre for Longitudinal Studies, University College London, London, United Kingdom; Division of Psychiatry, University College London, London, United Kingdom; Department of Statistical Sciences, University College London, London, United Kingdom; Department of Public Health and Nursing, Norwegian University of Science and Technology, Trondheim, Norway; Centre for Longitudinal Studies, University College London, London, United Kingdom; Centre for Longitudinal Studies, University College London, London, United Kingdom

**Keywords:** cohort studies, longitudinal studies, biobanks, polygenic scores, polygenic index

Key Features

- Birth cohort studies have enormous potential for use in genetically informed research across the health and social sciences; we introduce a resource to enable genetically informed research across multiple national birth cohort studies in the UK (1946–2001).
- These contain health and social data across life (from birth to older age), enabling longitudinal and cross-cohort genetically informed research. The Millennium Cohort Study additionally contains data on parents and offspring, enabling within-family analyses.
- In five cohorts born in 1946, 1958, 1970, 1989–90, and 2000–2, 36 603 individuals had harmonized, imputed, and quality-controlled genetic data from genotyping arrays covering 6.7 million common single-nucleotide polymorphism. The Millennium Cohort Study contains >6000 mother–offspring pairs and >3000 mother–father–offspring trios.
- Pseudonymized genomic data and polygenic indices/scores are available without charge to the global research community upon data application request.
- All data-access policies and materials, including application forms, can be found at https://cls.ucl.ac.uk/data-access-training.

## Data resource basics

### Background: the value of genomic data in multiple birth cohorts

**Figure 1.**
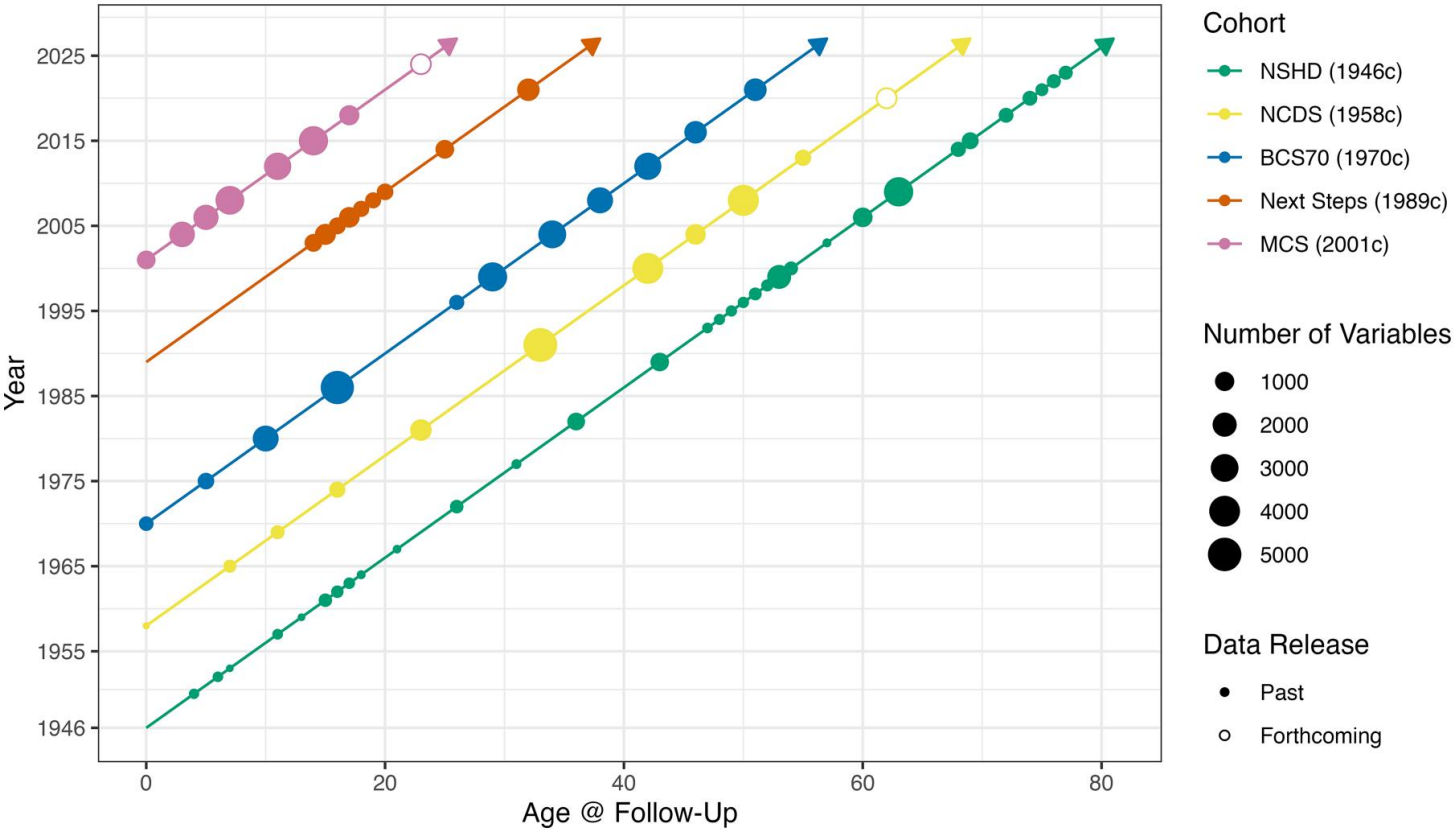
Phenotypic data in five British birth cohorts. The number of variables is indicative rather than comprehensive: as values cannot be compared across cohorts due to differences in variable derivation, they indicate the relative availability of variables across ages within each cohort.

Birth cohort studies have a rich history of contributing to science within and between disciplinary fields, notably health and social sciences [1–4]. Here, we introduce a curated resource comprising genomic data from multiple British birth cohort studies—longitudinal studies with rich data collected prospectively across life, each deliberately sampled to be nationally representative (Figure 1). We also outline our platform and steps to aid in cross-cohort harmonized analysis.

This paper provides information on the genotyping, imputation, and derived genetic data [polygenic indices (PGIs), also known as polygenic scores] for four national birth cohort studies initiated in 1946 (National Survey of Health and Development, 1946c) [5], 1958 (National Child Development Study, 1958c) [6], 1970 (British Cohort Study, 1970c) [7, 8], and the millennium (Millennium Cohort Study, 2001c) [9]. In addition, we include a cohort born in 1989–90, followed up from adolescence (Next Steps, 1989c). Genotyping was conducted for the main cohort participants in each study, enabling population-level longitudinal genetic analyses covering ≤78 years after birth. Furthermore, in the case of 2001c, the cohort participants’ co-resident (biological) mothers and fathers were also genotyped, enabling robust family-based genomic analyses [10, 11].

Phenotype data for these cohorts are extensive, including many thousands of variables, and have been described elsewhere; see [5–7, 9] and https://cls.ucl.ac.uk/cls-studies and https://nshd.mrc.ac.uk. Briefly, these studies have collected data on a wide range of domains, including physical and mental health, health behaviours (including, in a subset of cohorts, biomarkers and accelerometer data), psychosocial wellbeing, education, employment, cognition, personality, beliefs and attitudes, partnership formation and dissolution, and fertility across the life course. The studies have added further value through extensive data linkage to administrative and geographical data covering health, schooling, and local area factors (e.g. neighbourhood deprivation). For example, the cohorts are included in the UK Longitudinal Linkage Collaboration [12], which contains linkage to health records (e.g. hospital episode statistics, cancer registration data, maternity services, mental health records) and geographical datasets (e.g. NO_2_ and PM_2.5_ levels, green space, noise pollution). Phenotypic data have been measured regularly across the cohorts (Figure 1) from birth through adulthood and into older age (1946c and 1958c). Each is a ‘live’ cohort, with future data collections planned.

## Data collected

### Sample collection

Saliva samples were used for DNA collection in 2001c (14 years) and 1989c (32 years); in all other cohorts, blood samples were used, collected at 53 years (1946c), 44 years (1958c), and 46 years (1970c), respectively. Informed consent was obtained from participants and, for 2001c, their parents. Details of the data-collection protocols, responses, and predictors of response in 2001c have been previously described [13].

### Genotype data preprocessing

Genotype calling was performed by using GenomeStudio (v2.0, Illumina) and quality control (QC) was completed by using PLINK [14] 1.9 and 2.0. Samples were read into GenomeStudio (0–1.27% samples excluded) and mapped to a manifest file. Individuals were excluded if they had (i) >2% missing data (1.88%–3.50% of samples excluded); (ii) their genotype-predicted sex using X chromosome homozygosity was discordant with their reported sex (excluding females with an F value of >0.2 and males with an F value of <0.8) (0.18%–2.14% of samples excluded); (iii) they had excess heterozygosity (>3 SD from the mean) (0.36%–1.07% of samples excluded); (iv) for related individuals in 1946c, 1958c, 1970c, and 1989c, the King algorithm (king-cutoff 0.0884) was employed to identify and exclude one individual from each pair of closely related individuals (third degree or closer) (0.19%–0.59% of samples excluded). In 2001c, King was utilized to verify family relationships and rectify instances in which parents and children were incorrectly paired. In cases in which an individual could not be correctly matched to their family, that specific individual was removed from the dataset (0.12% of samples excluded). Duplicate samples were removed, retaining those with the higher genotyping rate.

We identified samples genetically similar to the European 1000 genomes samples by (i) merging the genotypes with data from 1000 genomes Phase 3, (ii) linkage disequilibrium pruning the overlapping single-nucleotide polymorphisms (SNPs) such that no pair of SNPs within 1000 bp had *r*^2^ > 0.20, and (iii) using an elastic net model to establish which of the 1000 genome superpopulations the samples are similar to [Africans (AFR), Admixed Americans (AMR), East Asians (EAS), Europeans (EUR), and South Asians (SAS)]. Although this method puts each sample into the nearest superpopulation, there are still ancestral outliers. The ancestral outliers can be removed based on principal components. We retain samples from all ancestries and provide a variable to capture this. Before imputation, SNPs with high levels of missing data (>3%), Hardy–Weinberg equilibrium *P* < 1e-6, or minor allele frequency of <1% were excluded.

The genetic data were then recoded as VCF files before uploading to the TOPMed Imputation Server, which uses Eagle2 to phase haplotypes and Minimac4 (https://genome.sph.umich.edu/wiki/Minimac4) with the TOPMed reference panel. The genome build was updated to hg38 by using LiftOver, implemented within the TOPMed server. This update applies to all data except 1989c, which already used the hg38 build. Imputed genotypes were then filtered with PLINK2.0alpha, excluding SNPs with an R2 INFO score of <0.8 and recoded as binary PLINK format. Proceeding with PLINK1.9, samples with >2% missing values, SNPs with at least two alleles, >3% missing values, Hardy–Weinberg equilibrium *P *< 1e-6, or a minor allele frequency of <1% were excluded (indels have not been excluded). In 1958c, five chips underwent QC separately and were combined after TOPMed imputation, where duplicated samples were removed, retaining those with a better genotyping rate (40% of samples excluded). QC checks were rerun on the combined sample, checking for related individuals across chips (0 samples excluded). For more details on samples that failed, please see Supplementary Table S1.

### Genomic data across the British birth cohorts

Table 1 displays the sample sizes for those who provided a biological sample that subsequently passed the imputation and QC procedures, as well as three possible denominators: (i) those who responded at the age of genotyping (i.e. provided some valid data such as via a survey questionnaire); (ii) those theoretically eligible for genotyping (e.g. those who had not died or emigrated), which corresponds to the target population of each cohort; and (iii) the total cohort (those who had ever responded). Response rates can be calculated by using either denominator—differences between studies may arise due to multiple reasons: differences in study scale (e.g. 1946c is smaller), the resourcing of each study, and the context in which each cohort has operated (e.g. 1989c was historically an education-focused study operating from within a UK government department, potentially adversely affecting willingness to provide DNA samples; secular declines in response rates have also occurred in social surveys). Response rates calculated by using participants (i) responsive at the age of genotyping (e.g. via survey response) and (ii) not known to have died or migrated at the age of genotyping as the denominator are as follows: 1946c: (92.1%, 64.8%), 1958c (68.2%, 40%), 1970c (65.2%, 33.8%), 1989c (21.5%, 10.2%), and 2001c (66.1%, 42.2%).

**Table 1.** Genomic data in British birth cohorts

Cohort	Age biological sample taken for genotyping (years)	*N* imputed + QC’d genetic data	*N* responsive at age of genotype^a^	*N* alive/in UK at age of genotyping^b^	*N* total birth cohort^c^	Genotyping array(s)	Post-imputation coverage
1946c NSHD	53 (then at 60–64, 69, 78)	2794	3035	4313	5362	Illumina Metabochip Illumina DrugDev Illumina NeuroX2	7 816 646
1958c NCDS	44 (then at 61–63)	6396	9377	15 971	18 558	Illumina 1.2 m Infinium 550 Infinium 550k Affymetrix v6 Illumina HumanQuad 660	7 545 708
1970c BCS	46	5598	8581	16 577	17 006	Illumina GSA array (v3)	8 640 849
1989–90c Next Steps	32	1568	7279	15 358	16 122	Illumina GSA array (v3)	8 084 945
2001c Millennium Cohort (members)	14	7836	11 859	18 575	19 517	Illumina GSA array (v1)	8 720 874
2001c Millennium Cohort (mothers)	28–65 (median 44)	7777	11 574	^d^	19 162	Illumina GSA array (v1)	8 720 874
2001c Millennium Cohort (fathers)	29–82 (median 47)	4634	10 414	^d^	19 162	Illumina GSA array (v1)	8 720 874

Note: Values may differ in future (e.g. if participants withdraw consent or greater genotyping coverage is obtained). Total *N*s are, for cohort members, *N* = 24 192, all participants: 36 603). For updated sample sizes, please see https://cls-genetics.github.io/.

a Those who provided some valid data (e.g. via a survey questionnaire).

b Those not known to have died or migrated at the time of biological sampling, including historical prior refusals (In Next Steps also excludes *n* = 22 participants in prison and includes only participants living in England per coverage of this study).

c Total birth cohort, including subsequent additions (e.g. during childhood sweeps). For 2001c, the total for mothers is lower than that of participants due to multiple births. For 1970c BCS, the total birth cohort number excludes those living in Northern Ireland who were sampled at birth but not followed up.

d Numbers alive at genotyping and in the UK are not provided for 2001c parents, as they are not the main cohort participants.

Response frequencies for each cohort and sweep can be obtained from the UK Data Service (UKDS) for 1958c (SN: 5560), 1970c (SN: 5641), 1989–90c (SN: 5545), and 2001c (SNs: 4683, 5350, 8156, 8172). SN: Series name.

### Within-family genomic data in 2001c

Genetic data were also obtained from the co-resident (biological) mothers and fathers of 2001c members at the same time as the participants. This collection of family genomic data enables 2001c to be used for robust family-based genetically informed analyses (see below). These samples (Table 2) were processed at the same time and in the same way as those for the participants, being genotyped on the same Illumina GSA array (v1) chip: 7777 study mothers and 4634 study fathers provided samples that passed imputation and QC. Biological sample provision was nested within families, meaning that many of the participants who provided a sample also had a mother or father who provided a sample, and 40% had data from a complete family trio. This resulted in 6426 offspring + mother duos, 3802 offspring + father duos, and 3117 offspring + mother + father trios.

**Table 2.** Genomic family data in the Millennium Cohort Study: sample sizes for each familial configuration.

	*N*
Millennium Cohort members (offspring)	7836
Millennium Cohort mothers	7777
Millennium Cohort fathers	4634
Millennium Cohort member (offspring)/mother duos	642 6
Millennium Cohort member (offspring)/father duos	380 2
Millennium Cohort member (offspring)/mother/father trios	3 117

Sample sizes are shown for those with valid imputed genetic data who passed QC. Values may differ in future (e.g. if participants withdraw consent or greater genotyping coverage is obtained). For updated sample sizes, please see https://cls-genetics.github.io/docs/MCS.html.

### Polygenic index generation

PGIs have been calculated for health and social traits (Table 3). We plan to periodically update these in future as genome-wide association studies (GWAS) for additional traits, more powerful GWAS for existing traits, and as new PGI scoring methods become available. For an up-to-date list of available PGIs, please see the Centre for Longitudinal Studies (CLS) genomics page at https://cls-genetics.github.io. The current release of PGIs is generated by using a clumping and thresholding approach in the software package PRSice2 (v2.0) [15]. We used an additive scoring method that sums trait-associated variants in each cohort for each participant, weighted by their effect size estimates from GWAS summary statistics (i.e. the number of effect alleles multiplied by the beta or log odds ratio for each SNP). PGIs have been calculated in each dataset for each individual at a range of *P*-value thresholds including genome-wide significance (*P* < 5e-08). ​Each PGI has been generated twice in each cohort—first by using the maximum number of SNPs that pass QC in each cohort and second by using a set of SNPs (*N* = 6 702 716) that are harmonized across all cohorts. Future planned releases of PGIs will include those derived by using advanced methods such as LDpred2. In addition to the CLS PGI repository, 1958c, 1970c, and 2001c are included in the SSGAC Polygenic Index Repository v2 [16].

**Table 3.** Polygenic indices curated in British birth cohorts.

Anthropometrics	Birthweight
	Body fat distribution
	Body mass index (childhood)
	Body mass index (adulthood)
	Grip strength
	Height
	Waist circumference
Brain structure and cognition	Alzheimer’s disease
	Cognition
	Hippocampal volume
	Parkinson’s disease
Health behaviours	Age at initiation of smoking
	Alcoholic drinks per week
	Cigarettes per day
	Diet
	Substance abuse
Mental health	Anxiety
	Attention Deficit Hyperactivity Disorder (ADHD)
	Autism spectrum disorder
	Bipolar disorder
	Depressive symptoms
	Externalizing problems
	Major depressive disorder
	Schizophrenia
Personality	Agreeableness
	Conscientiousness
	Extraversion
	Openness to experience
	Neuroticism
Physical health	Age at menopause
	Asthma
	Blood pressure
	Coronary artery disease
	C-reactive protein
	Fasting glucose
	HbA1c
	Hypertension
	Rheumatoid arthritis
	Type 1 diabetes
	Type 2 diabetes
Social outcomes	Education
	Household income
	Human longevity
	Parental lifespan

Traits included may increase in future (e.g. as underlying GWAS studies increase in size). For updated information, please see https://cls-genetics.github.io.

## Data resource use

Birth cohorts played key early roles in understanding the genetic and environmental aetiology of common diseases [17]. In recent years, there has been an explosion of interest in and use of large-scale biobanks drawn from non-representative samples such as UK Biobank (*N* = 500 000) and, latterly, Our Future Health in the UK (target *N* = 5 million) and All of Us in the US (target *N* = 1 million). How can these smaller cohorts add value in the context of large biobanks and the broader scientific literature?

First, longitudinal cohort studies enable study across the entire lifespan; follow-up is from birth to older ages. This allows the investigation of genetically informed research with a life-course framework. For example, do genetic contributions to traits differ across life? Recent work has investigated this for body mass index [18, 19] and blood pressure [20], but, given the paucity of life-course datasets combined with genetics, it remains unclear how such patterns differ for other health or social traits, or how gene–environment interactions differ across life. Where power is insufficient in a given cohort, cohorts can be pooled [21].

Second, longitudinal cohort studies are interdisciplinary resources; their rich health and phenotypic data enable the testing of many research questions that are impossible to answer with the limited cross-sectional questionnaire data typically available in large biobanks. This will allow new evidence to be brought to light on traits that are well studied in cohort studies, such as using genetic variants as exposures, confounding variables, or instrumental variables to mitigate reverse causality/confounding [22]. For those more familiar with larger-scale biobanks, it will enable the study of phenotypic data that are typically unmeasured or measured poorly in medically oriented biobanks, e.g. child development, personality, cognitive ability, socioeconomic measures across life, and wellbeing. The data can also be used to better understand the results from large biobanks that generally use very coarse measurements, e.g. of socioeconomic position [23]. Prospective measurement avoids issues relating to retrospective recall bias.

Third, using multiple longitudinal cohorts allows comparative research across time, enabling the scientific study of generational change [4, 24]. Several phenotypes, including smoking, body mass index (BMI), mental health, and education, have dramatically changed across the latter half of the twentieth century. For many outcomes, genetic variants’ absolute and relative importance may depend on the societal context in which genes are expressed (e.g. as has been suggested for education [25, 26] or BMI [27]). For example, a recent study using this resource found markedly larger associations between genetic liability to obesity and observed BMI in more recently born children (2001c versus earlier-born cohorts) [27]. Though the geographic span and ethnic diversity of the cohorts differ slightly, simple restrictions can be made to make them comparable (e.g. in all birth cohorts, focus on cohort members of White ethnicity from mainland Britain to reflect the ethnic make-up and sampling frame used for 1946c). Providing data that have been imputed and undergone QC across the cohorts means that these data can be analysed consistently and efficiently.

Fourth, their sampling design and national representation aid greater generalizability to the target population and understanding of the broader role of selection bias in genetically informed research than is possible in other large-scale biobanks. Though 1989c and 2001c used complex sampling designs with oversamples of disadvantaged groups, design weights are available to account for this. While attrition remains a concern across longitudinal studies (as does non-response in cross-sectional studies [28]), there is increasing evidence that the rich data obtained throughout early life in cohorts can reduce bias due to attrition in analyses [29–31]. Future work could use these datasets to test the importance of selection bias in larger-scale studies empirically.

Finally, the youngest of the cohorts that we include contains genetic data on family trios (mothers, fathers, and cohort member offspring). This enables the separation of direct and indirect genetic effects on outcomes through adjustment for parental genotypes [10, 11] and modelling of parental assortment [22, 32–34].

## Strengths and weaknesses

Strengths include the consistent approach to data QC and imputation, and features of the data detailed above, including rich data across life, multiple generations, a nationally representative sampling frame, and family-based genomic data (in 2001c).

Weaknesses of the genomic data resources in the British birth cohorts include non-response. DNA was not collected at birth and attrition rates have differed across cohorts and over time. Those lost to follow-up in these cohorts tend to be in worse health, from disadvantaged socioeconomic backgrounds, and from minority ethnic groups [29, 30, 35, 36]. However, bias can be mitigated by using principled approaches to handle missing data, such as multiple imputation, full information maximum-likelihood, or analytical weights. A key benefit of cohort data is the availability of information on cohort members before they attrit; recent work shows that these data can substantially reduce non-response bias [29–31]. We provide users with guidance to use such approaches in their analyses [37] and, in future, will provide inverse probability weights for genetically informed analyses.

As in other harmonization initiatives, information loss typically results when analysing multiple cohorts together using the same SNP coverage, which is limited to the lowest common denominator across included cohorts. We provide SNP data for each cohort and harmonized SNP data that are restricted to overlapping SNPs across all cohorts to enable more reliable comparisons across cohorts. High coverage across the harmonized SNP data results in little loss of information and statistical power. For example, by using a genome-wide significant PGI for educational attainment and observed education at age 33 years in 1958c, the incremental *r*^2^ of a PGI (above sex and principal components) derived from SNPs harmonized across the cohorts is 9.17% compared with 9.21% for PGIs derived from unharmonized SNPs. Conducting sensitivity analyses in cohorts without this restriction (i.e. the largest available coverage without any restrictions) may inform us of the potential implications.

Finally, the ancestral diversity of adult cohorts born before migration into Britain in the later twentieth century is limited (i.e. 1946c, 1958c, and 1970c). However, 2001c and 1989c included oversampling of ethnically diverse areas, enabling genetically informed analyses across diverse ancestry groups [13]; this is evident when compared with the 1000 genome samples. Pooling analyses across multiple cohorts may mitigate power issues if some ancestral samples are insufficiently large in a single cohort.

## Data resource access

Pseudonymized data are freely available to the research community upon approval of a data application request. Access is governed by the CLS Data Access Committee (CLS DAC). Researchers may also apply to use stored biological (e.g. blood, saliva) samples for new assays. All data-access policies and materials, including application forms, can be found at https://cls.ucl.ac.uk/data-access-training. Full genetic data are made available upon application to CLS; polygenic indices will be made available via the UK Data Service.

A register of approved genetic projects is also maintained on the CLS data-access page. The CLS DAC evaluates all requests following the principles and criteria outlined in the CLS Data Access Framework, ensuring that data are shared responsibly and securely, and accessed only by worldwide bona fide researchers with due consideration to relevant ethical issues. Researchers should carefully review the CLS DAC data-access guidelines and submit a completed application form to clsdata@ucl.ac.uk. Subject to their development, future access may be via a Trusted Research Environment. For access to 1946c, see skylark.ucl.ac.uk. General research enquiries can be made to david.bann@ucl.ac.uk.

## Ethics approval

Ethical approval was obtained in each study: 1946c (North Thames Multicentre Research Ethics Committee: reference 98/2/121 and 07/H1008/168), 1958c (South East Multi-centre Research Ethics Committee: ref 01/1/44), 1970c (South East Coast—Brighton & Sussex: ref 15/LO/1446), 1989c (East of England—Cambridge Central Research Ethics Committee: ref 22/EE/0052), 2001c (London-Central REC: 13/LO/1786).

## Supplementary Material

dyaf141_Supplementary_Data
