# Supplementary material for: Data Resource Profile: Genomic data in multiple British birth cohorts (1946–2001)—linkage with health, social, and environmental data from birth to old age
Source: Int J Epidemiol. 2025 Aug 18;54(5):dyaf141. doi: 10.1093/ije/dyaf141 (PMC12360837; doi:10.1093/ije/dyaf141)
Supplement: dyaf141_Supplementary_Data [file dyaf141_supplementary_data.docx]

**Supplementary Table S1. Sample sizes and those excluded at each stage of the quality control (QC) process.**

| **QC Step** | **1946c** | **1958c** | **1970c** | **1989c** | **2001c** |
| --- | --- | --- | --- | --- | --- |
| Starting samples | 2,912 | 13,738 | 5,905 | 1,683 | 21,566 |
| Read into GenomeStudio | 2,912 | 13,738 | 5,830 | 1,681 | 21,556 |
| Individual-level exclusions (e.g. consent issues/ withdrawal) | 0 | 67 ^a^ | 0 | 0 | 348 |
| >2% missing genotype rate per individual | 102 | 204 ^a^ | 136 | 57 | 667 |
| Mismatched sex | 20 | 20 ^a^ | 15 | 36 | 86 |
| Excess heterozygosity | 12 | 61 ^a^ | 46 | 18 | 78 |
| Mismatched samples based on KING | NA | NA | NA | NA | 26 |
| Related families ^b^ | NA | NA | NA | NA | 212 |
| Remaining samples for merge ^c^ | NA | 10,829 | NA | NA | NA |
| Duplicate samples excluded on merge ^c^ | NA | 4,358 | NA | NA | NA |
| Individual-level exclusions after merge ^c^ | NA | 52 | NA | NA | NA |
| King related individuals | 1 | 23 | 35 | 5 | NA |
| European (using 1000 Genomes Phase 3 labels) | 2,731 | 6,324 | 5,361 | 1,272 | 17,460 |
| **Total** | 2,777 | 6,396 | 5,598 | 1,568 | 20,247 |

Note: values may differ in future (e.g., if participants withdraw consent or greater genotyping coverage is obtained). For updated sample sizes, please see: [https://cls-genetics.github.io/](https://cls-genetics.github.io/docs/intro.html)

^a^ for 1958c individual-level exclusions, multiple samples were removed per individual removed across the individual chips.

^b^ indicates steps applicable only to the 2001c.

^c^ indicates steps applicable only to the 1958c.

NA values indicate QC steps not applicable to specific cohorts.
